# Supplementary material for: Content-rich biological network constructed by mining PubMed abstracts
Source: BMC Bioinformatics. 2004 Oct 8;5:147. doi: 10.1186/1471-2105-5-147 (PMC528731; doi:10.1186/1471-2105-5-147)
Supplement: Additional File 2 — The original results of the above study (non-essential files are deleted to keep the file size under the limit set by BMC bioinformatics). [file 1471-2105-5-147-S2.bz2 › chilibotAdditionalFile2/dip10/378ID9927421E9791/html/NGFR_NTRK2.html]

 


 **NGFR** and **NTRK2** 
  
Found 102 abstracts in PubMed, retrieved 10.  
 

 What does Google say? 
 PDF only 
| .edu only 

---

**Interactive relationship** (e.g. stimulation, inhibition, etc)

**Neutral relationship**- Although neurotrophins did not regulate p75NTR  [ **NGFR** ]  processing, the alpha and gamma secretase mediated cleavage of p75 is modulated by receptor tyrosine kinases Trks TrkA and TrkB  [ **NTRK2** ]  but not TrkC.  Ref: 12843241 J Neurosci, 2003

**Non-interactive relationship** (e.g. studied together, co-existance, homology, etc.)

- The specimens were analyzed immunohistochemically for neurotrophins nerve growth factor, brain derived neurotrophic factor, neurotrophin 3, neurotrophin 4 5 and their receptors TrkA, TrkB  [ **NTRK2** ] , TrkC, p75NTR  [ **NGFR** ] .  Ref: 11442674 Int J Urol, 2001
- Neurotrophin signaling is mediated by Trk family tyrosine kinases such as TrkA, TrkB  [ **NTRK2** ]  and TrkC, as well as by the pan neurotrophin receptor p75NTR  [ **NGFR** ] .  Ref: 11836532 Nat Neurosci, 2002
- Sections of pancreatic cancer and normal pancreatic tissue were examined by immunohistochemistry for the expression of the neurotrophins NGF, BDNF, NT 3, NT 4, and their receptors TrkA, TrkB  [ **NTRK2** ] , and TrkC, as well as the low affinity receptor, p75NTR  [ **NGFR** ] .  Ref: 11561004 J Histochem Cytochem, 2001
- Activation of the TrkB  [ **NTRK2** ]  and the p75NTR  [ **NGFR** ]  by the addition of BDNF facilitates neuronal commitment and marked neurite genesis.  Ref: 12589813 Biochem Biophys Res Commun, 2003
- The neurotropins are survival and differentiation factors in the nervous system, acting via high affinity receptors of the trk protooncogene family and the low affinity p75 nerve growth factor receptor  [ **NGFR** ] , and have also been described in the rodent ovary, where changes in NT4 TrkB  [ **NTRK2** ]  gene expression have been detected at the time of primordial follicle formation.  Ref: 11836338 J Clin Endocrinol Metab, 2002
- In contrast, the expression of TrkB  [ **NTRK2** ]  was down regulated and the expression of p75NTR  [ **NGFR** ]  was up regulated in prostate cancer after hormonal therapy.  Ref: 11442674 Int J Urol, 2001
- We have used immunofluorescence techniques with a panel of antibodies against known neurotrophin receptors trk A, trk B  [ **NTRK2** ] , trk C, p75NTR  [ **NGFR** ]  to map the locations of these receptors in the developing neuromuscular system of mice from our neurotrophin 3 NT 3 knockout colony.  Ref: 12141445 Int J Dev Biol, 2002
